# Design and Acoustic Performance Research of Underwater Acoustic Absorption Metamaterials

**DOI:** 10.3390/ma18225075

**Published:** 2025-11-07

**Authors:** Guangqi Dong, Fengmin Wu

**Affiliations:** Department of Physics, School of Science, Harbin University of Science and Technology, Harbin 150080, China; d15776650548@163.com

**Keywords:** underwater acoustic absorption, metamaterial, broadband absorption, finite element simulation, low-frequency noise control

## Abstract

This study designs an underwater acoustic absorption metamaterial based on a multi-cavity diaphragm structure. The acoustic performance is carefully modeled and examined through simulations in COMSOL Multiphysics finite element software (v.6.1). First, a multilayer periodic unit model consisting of a main cavity and sub-cavities is constructed. A corresponding acoustic-structure coupled finite element model is established by incorporating diaphragm thickness and pre-tension parameters. The frequency domain analysis method is then employed to simulate sound wave transmission and resonance absorption within the structure, calculating the relationship between the acoustic absorption coefficient and frequency. Based on parametric sensitivity analysis, the study examines the influence of key parameters, including main cavity depth, slit width, sub-cavity depth, diaphragm thickness, and pre-tension, on acoustic absorption performance. The mechanisms by which these parameters regulate the absorption peak and bandwidth are revealed. The simulation results show that this metamaterial provides effective broadband acoustic absorption from 200 Hz up to 3000 Hz. The effective bandwidth with an absorption coefficient (α > 0.5) reaches 770 Hz, with a maximum absorption peak of 0.96 and an average absorption coefficient of 0.74, indicating excellent low-frequency underwater acoustic absorption capability. This study provides theoretical foundations and design guidelines for underwater noise control and related engineering applications.

## 1. Introduction

With the continuous growth of marine resource development, naval equipment construction, underwater communication, and other fields, the complexity of the underwater acoustic environment and noise pollution problems have become increasingly prominent [1,2]. A large amount of low-frequency noise generated by activities such as ship navigation, industrial equipment operation, and marine oil and gas exploitation interferes with the normal behavior patterns of marine organisms; it also seriously affects the performance of underwater communication and detection systems [3,4]. Therefore, developing sound-absorbing materials with superior performance, compact structure, and suitability for complex underwater environments has become an important direction in current underwater acoustics research [5].

Traditional underwater acoustic absorption materials such as rubber, polyurethane foam, and porous polymers usually rely on viscous loss and thermal dissipation mechanisms to achieve acoustic energy absorption [6]. Although such materials have a certain acoustic absorption capacity in the medium and high-frequency bands, they often perform poorly in the low-frequency band. In addition, to achieve effective absorption, these materials often need to be quite thick, which can result in bulky designs and reduced flexibility or compatibility with other components [7,8]. This is especially problematic in systems like ships and submarines, where space is limited and maintaining quality is important [9]. Therefore, achieving effective absorption in a wide frequency band, especially the low-frequency band, under the condition of limited thickness, is one of the key bottlenecks in the development of underwater acoustic absorption materials.

In recent years, advances in artificial structural materials have enabled the development of acoustic metamaterials. These materials achieve extraordinary acoustic properties, such as negative effective density or bulk modulus, through precisely designed microstructures, offering new solutions to overcome the acoustic absorption limitations of traditional materials [10,11]. In particular, metamaterial structures based on local resonance mechanisms can excite strong resonance at the subwavelength scale, showing excellent low-frequency acoustic absorption characteristics [12]. Besides, the expansion and regulation of the absorption frequency band can be achieved by introducing configuration designs such as multi-scale structures, composite damping layers, and rigid backplanes. Such metamaterials with high designability and integrated functions bring broad research prospects to the field of underwater acoustic absorption [13].

In the domain of underwater noise control, the design and performance optimization of acoustic absorption materials have always been one of the core issues of research. Traditional absorbing materials mainly rely on the viscous damping and thermal dissipation mechanisms of porous media to achieve sound energy attenuation [14]. Zarastvand et al. [15] attempted to construct multi-layer composite structures to enhance acoustic absorption capacity in the low-frequency band. These structures included superimposing elastic layers or rigid plates on the outside of traditional porous materials to increase sound wave residence time and interface reflection loss. Wu et al. [16] proposed that the composite structure formed by an air interlayer and a porous sound-absorbing layer could expand the effective absorption frequency band and improve low-frequency performance. However, they also introduced new problems such as increased structural complexity and reduced environmental adaptability. In addition, Yang et al. [17] pointed out that adjusting porosity and interlayer damping parameters could improve acoustic absorption performance to a certain extent. Nevertheless. the overall improvement was limited, making it difficult to break through the trade-off between physical thickness and performance.

Against this background, the rise of acoustic metamaterials provides a new idea for solving underwater acoustic absorption problems. Jang & Song [18] proposed the concept of locally resonant acoustic metamaterials, proving that they could achieve strong sound wave regulation even with small structural sizes. Subsequently, more and more studies have been carried out around resonance cavity structures, diaphragm resonators, maze-like channels, etc., to explore the regulation mechanisms of low-frequency sound waves in depth. For the underwater environment, Xu et al. [19] constructed a locally resonant structure applicable to underwater propagation media. This confirmed that it could gain obvious absorption peaks in the low-frequency band of 400~800 Hz. At the same time, directional tuning of the absorption frequency could be realized by adjusting structural parameters. Xie et al. [20] developed a multi-cavity parallel structure, which could obtain multiple absorption peaks with limited thickness and demonstrated certain broadband performance. These studies prove that metamaterials show great potential in the underwater acoustic absorption field through precise regulation of structural geometry. However, existing studies mostly focus on the structural design level; discussions on the adaptability between material selection and underwater working conditions (such as high pressure, saltwater corrosion) are relatively few; and a systematic design method for actual underwater applications has not yet been formed.

Over the years, some scholars have also introduced Micro-perforated Panels (MPP) or damping interlayers into metamaterial structures to expand the absorption frequency band. Li et al. [21] found that micro-perforated arrays combined with back cavity structures could effectively absorb medium and high-frequency sound waves, especially suitable for scenarios with high sound source frequencies like shipborne equipment. Meanwhile, adding high-damping rubber materials as intermediate interlayers helped to improve broadband energy dissipation capacity and alleviate problems such as overly sharp resonance peaks or excessively narrow frequency bands [22,23,24]. However, the coupling complexity between structural parameters increased remarkably, leading to greater challenges in the design optimization process. In addition, acoustic metamaterials, as an emerging type of artificial structural material, have attracted widespread attention due to their extraordinary acoustic regulation capabilities at the sub-wavelength scale. Liao et al. [25] systematically reviewed the main theoretical models, structural types, preparation methods, and application scenarios of acoustic metamaterials. They pointed out that multi-scale coupling and multi-functional integration were important future development directions. Particularly in underwater acoustics, Dong et al. [26] summarized the research progress of underwater acoustic metamaterials. They emphasized the challenges and opportunities they faced in extreme environments such as high hydrostatic pressure and strong corrosion. Failla et al. [27] further explored the scientific and technical issues existing in multi-physics field design from the perspective of coupling between elastic waves and acoustic waves. In addition, the design concept of acoustic metamaterials is also constantly undergoing cross-domain integration. For example, Xu et al. [28] applied them to transdermal drug delivery systems in biomedicine, demonstrating the potential of acoustic structures in energy focusing and control. Comandini et al. [29] proposed an integrated design paradigm from the perspective of architectural materials, providing new ideas for the structure-function integration of acoustic metamaterials. These studies collectively show that acoustic metamaterials are gradually moving from basic concept exploration to practical engineering applications. At the same time, their design methods are becoming increasingly diverse and systematic.

To sum up, existing underwater acoustic absorption materials have different degrees of deficiencies in structural complexity, performance stability, and low-frequency absorption efficiency. The development of multifunctional composite acoustic absorption structures, designed around metamaterial principles, holds important promise for advancing underwater acoustic technologies. Based on existing research, this study intends to design a new type of acoustic absorption metamaterial structure integrating local resonance cavities, micro-perforated structures, damping interlayers, and backplane reflective surfaces. It deeply explores this structure’s acoustic mechanism and performance relationship, and verifies its effectiveness in low-frequency broadband acoustic absorption by combining specific material parameters and underwater simulation environments. Thus, it can provide theoretical support and structural reference for subsequent engineering of underwater acoustic stealth applications.

The research innovation is mainly reflected in three aspects. First, by conducting an integrated composite design of local resonant cavities, micro-perforated array layers, damping interlayers, and backing rigid plates, a multi-layer coupled unit with gradient impedance characteristics is constructed. This breaks through the limitations of traditional single structures in terms of bandwidth and low-frequency efficiency. Second, a multi-cavity coupling and pre-tensioned film adjustment mechanism is introduced, enabling active regulation of multiple absorption peaks in the 200–3000 Hz frequency band within a limited thickness. Third, practical environmental constraints such as underwater high pressure and corrosion are fully considered in the design process. The selected materials and modular structural design significantly enhance the engineering application potential of this structure, providing a high-performance and practical solution for underwater equipment noise control.

## 2. Research Methodology

### 2.1. Structural Design of Underwater Acoustic Absorption Metamaterial

The underwater acoustic absorption metamaterial structure designed in this study is constructed based on multi-layer periodic composite units. The periodic unit extends infinitely in a two-dimensional periodic arrangement within the plane formed by the *x*-axis and *y*-axis. However, in the *z*-axis direction (i.e., the thickness direction), it is formed by stacking multi-layer functional materials, thus constituting the entire metamaterial panel. It aims to achieve efficient absorption of incident sound waves and suppression of reflection within the target frequency band. The overall structure adopts a two-dimensional planar arrangement, containing array-distributed acoustic absorption units to enhance the sound waves’ local resonance and interference effects. Each metamaterial unit is composed of multiple functional layers stacked together, encompassing a panel layer, a damping layer, a supporting substrate layer, and a backing rigid plate, forming a typical “soft-hard-soft” multi-interface structure. This effectively expands the energy coupling range of low-frequency sound waves.

**Figure 1:**
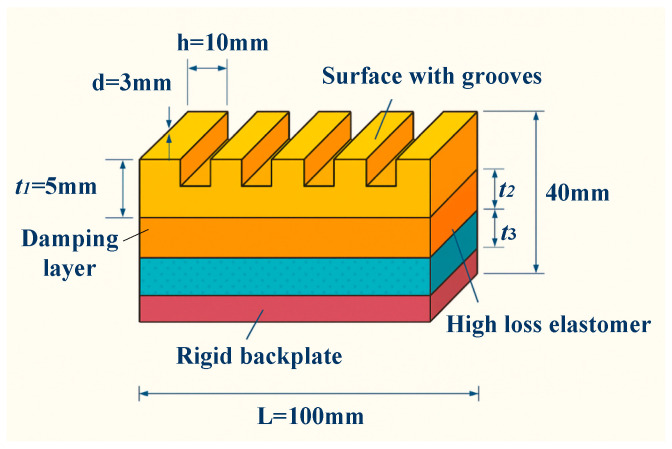
Structural schematic diagram of underwater acoustic absorption metamaterial.

The geometric configuration of the structure is displayed in Figure 1, with a design size of L = 100 mm in both length and width, and H = 40 mm in the height direction. The specific thicknesses of each functional layer are as follows. The surface wave-absorbing, middle-damping, and bottom substrate layers have a thickness of t_1_ = 5 mm, t_2_ = 10 mm, and t_3_ = 25 mm. Among them, the damping layer is made of high-loss elastomer, which has good energy dissipation capacity; the substrate layer uses high-strength polymer foam to ensure overall stability and processability. The surface of the wave-absorbing layer is designed with a periodic groove array, with a groove spacing of d = 10 mm and a depth of h = 3 mm, which can induce multiple reflections and interference of incident waves, thereby increasing the energy density per unit volume.

The noise reduction logic behind the structural design is based on three mechanisms. First, local resonance: the internally filled structure induces low-frequency sound waves to resonate with local media, thus achieving absorption peaks at specific frequency points. Second, multiple reflection paths: the irregular boundaries and periodic groove arrays inside the structure can extend the sound wave transmission path and increase energy dissipation. Third, impedance matching and interface regulation: the gradient design of soft and hard interfaces reduces the sound wave reflection coefficient and improves the effective coupling rate of incident energy. These mechanisms have been initially verified in acoustic simulations, showing good low-frequency absorption performance.

Moreover, to enhance the feasibility of practical applications, the design fully considers the pressure stability and material corrosion resistance in the underwater environment. The selected materials all have high pressure resistance and watertightness. The structural processing adopts modular unit assembly, facilitating fast deployment and replacement at engineering sites. The overall structure not only has good scalability and maintainability but also achieves an ideal balance between acoustic absorption per unit area and structural weight. Thus, it offers theoretical and engineering support for its application in scenarios such as hull coating and underwater equipment shielding.

### 2.2. Material Selection and Composition Analysis

Based on the above multi-layer periodic composite unit design, the underwater acoustic absorption metamaterial constructed in this study adopts a “soft-hard-soft” multi-interface laminated structure. The purpose is to achieve efficient absorption of incident sound waves and suppression of reflection within the target frequency band. The reasonable selection and performance matching of materials for each layer are key to ensuring the acoustic performance of the overall structure. As the first interface of the metamaterial unit, the panel layer undertakes the functions of initial impedance matching of sound waves and excitation of local resonance. Considering the high acoustic impedance of the underwater environment and the requirements for mechanical stability, the panel layer is made of high-elastic polymer film, which has moderate elasticity and density. It can induce local vibration resonance while playing a role in flexible adjustment among multi-layer interfaces, promoting energy coupling of sound waves between layers.

The damping layer is placed between the panel and the supporting substrate layers, with its main function being to absorb vibration energy and achieve sound wave dissipation. This study selects a polymer elastomer material with high internal friction to enhance low-frequency acoustic absorption capacity. This material has moderate density and acoustic impedance, along with strong damping properties, which help convert sound wave energy into thermal energy loss and substantially improve absorption efficiency.

As the structure’s rigid support and acoustic adjustment layer, the supporting substrate layer needs to have a high elastic modulus and density to effectively restrict the vibration of the panel layer and form an acoustic path with multi-cavity coupling. In terms of materials, this study uses hard polymer composites commonly used underwater. Their excellent mechanical properties ensure structural stability, while their appropriate acoustic characteristics are conducive to multiple reflections of sound waves and coupling of local resonance.

The backing rigid plate is the last interface of the metamaterial unit, mainly responsible for reflecting unabsorbed sound waves to form a standing wave effect, promoting the accumulation of sound wave energy in the absorption and damping layers. Considering the requirements for corrosion resistance and mechanical strength in underwater use, stainless steel or aluminum alloy with a thickness of approximately 3 mm is selected as the material for the backing rigid plate. It has both high density and rigidity, ensuring the mechanical integrity and long-term stability of the structure. Table 1 summarizes the key physical and acoustic parameters of each layer of materials, including density (ρ), sound velocity (c), acoustic impedance (Z = ρc), and elastic modulus (E). These can provide an accurate basis for subsequent theoretical analysis of multi-cavity coupling mechanisms and simulation modeling.

### 2.3. Mechanism of Multi-Cavity Coupled Acoustic Absorption

The constructed underwater acoustic absorption metamaterial achieves broadband absorption of sound waves in the mid-low frequency band through the integration of periodic “soft-hard-soft” multi-layer structures and multi-cavity units. This absorption process involves the synergistic effect of multiple acoustic mechanisms, mainly including local resonance, Fabry–Pérot cavity resonance, multi-cavity coupling interference, and equivalent mass-spring system oscillation. When sound waves of a certain frequency incident from water to the surface of the metamaterial, they first excite local resonance in the flexible panel layer. The local vibration of the structure in the vertical direction can be approximated as a simplified mass-spring system, whose resonance frequency is given by Equation (1):

(1) $$f_{r}=\frac{1}{2\pi }\sqrt{\frac{k_{eff}}{m_{eff}}}$$

$k_{eff}$ refers to the equivalent stiffness of the system; $m_{eff}$ represents the equivalent mass of the panel layer and the attached damping layer. By adjusting the material thickness, tension, and arrangement, the resonance response of the structure can be effectively controlled, enabling it to strongly couple with sound waves within the target frequency band.

Further, multiple closed or open-air cavities or filled cavities are formed between the panel and the rigid backplane. Under specific conditions, these cavities satisfy the Fabry–Pérot resonance relationship, causing sound waves to undergo standing wave enhancement inside the cavities. Their resonance frequency $f_{n}$ is expressed as:

(2) $$f_{n}=\frac{nc}{2d},n=1,2,3,\ldots$$

*c* means the sound velocity in the medium; and *d* denotes the effective length of the cavity. The periodic array composed of multiple cavities forms a dispersive structure, and different cavities generate multiple absorption peaks, thus exhibiting broadband acoustic absorption characteristics.

**Figure 2:**
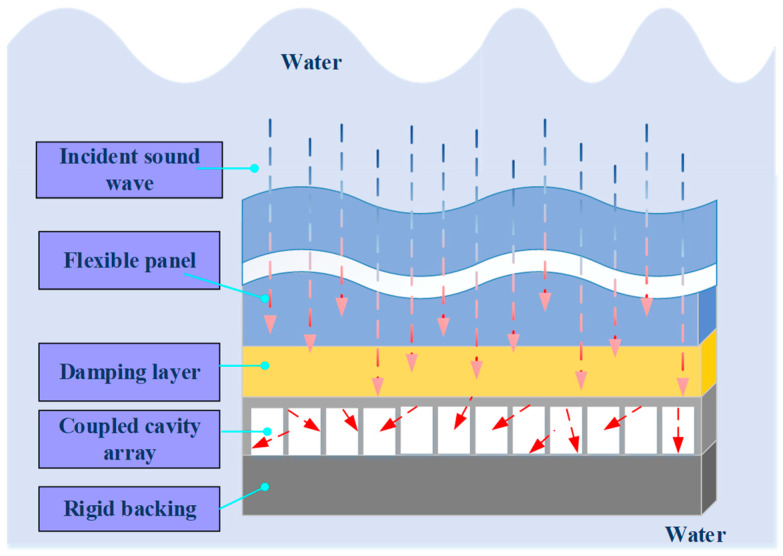
The internal sound energy propagation path and resonance mode of the underwater acoustic absorption unit structure.

To describe the above coupling process more clearly, Figure 2 shows the internal sound energy propagation path and resonance modes of the unit structure. The flexible panel layer forms a vibration source after responding to sound pressure, which conducts downward through the damping layer to excite multiple resonant cavities. Sound waves reflect multiple times between different cavities, forming high-intensity interference and standing wave regions, and realizing spatial superposition and dissipation of energy.

To quantitatively simplify the propagation and loss behavior of sound waves in the structure, this study introduces an equivalent circuit model to model the unit structure. In this model, the flexible panel is regarded as a mass unit *M*, the damping layer as a damping unit *R*, and the cavity as an elastic unit *C*, forming an M–R–C series oscillation circuit. The parallel combination of multiple unit circuits constitutes the overall absorption system, whose frequency response function can be written as:

(3) $$H(f)=\frac{1}{\sqrt{(1-(\frac{f}{f_{r}}{)}^{2}{)}^{2}+(2\zeta \frac{f}{f_{r}}{)}^{2}}}$$

ζ represents the damping ratio, and $f_{r}$ denotes the main resonance frequency. By changing the values of various parameters in the equivalent circuit, the response intensity and acoustic absorption bandwidth of the structure in different frequency bands can be adjusted. The core advantage of the structure’s multi-cavity coupling mechanism lies in that its multiple functional layers can excite resonance behaviors in diverse frequency bands, respectively. in contrast, the size regulation of the cavities and the optimization of the array period can further improve the distribution density and spatial coverage of resonance frequencies.

**Figure 3:**
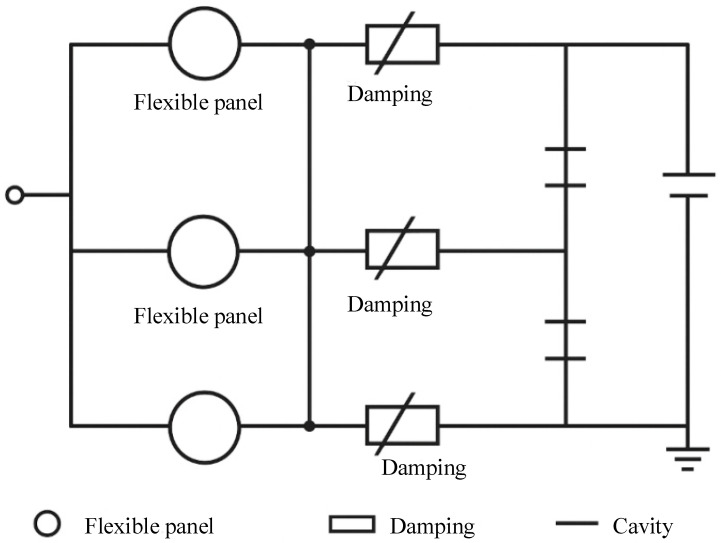
Equivalent circuit diagram of the unit structure.

To express this equivalent circuit model more intuitively, Figure 3 shows its schematic diagram. In the diagram, the flexible panel is regarded as a mass unit, the damping layer as a damping unit, and the cavity as a compliance unit; these three units form a series oscillation circuit.

### 2.4. Simulation Verification and Parameter Sensitivity Analysis

During the simulation modeling process, this study uses the Pressure Acoustics module and Solid Mechanics module in COMSOL Multiphysics software (v.6.1) to conduct acoustic-structural coupling finite element analysis. The coupling process between the acoustic field and the solid structure is established based on continuum mechanics and acoustic control equations. In the acoustic domain, the sound pressure disturbance in the fluid medium satisfies the linearized wave equation, and its frequency-domain form is:

(4) $$\nabla \cdot \left(-\frac{1}{\rho }\nabla p\right)-\frac{\omega^{2}}{\rho c^{2}}=0$$

p represents the sound pressure; ρ means the medium density; *c* is the sound speed; ω refers to the angular frequency. Equation (4) is a frequency-domain control equation derived based on the assumption that the sound pressure has a harmonic form: $p\left(r,t\right)=\hat{p}(r)e^{jwt}$; it is used to describe the propagation of the steady-state acoustic field. In the solid structure domain, the elastic vibration of the structure is described by the linear elastic kinetic equation:

(5) $$\rho_{s}\frac{\partial^{2}u}{\partial t^{2}}-\nabla \cdot \sigma =F$$

u denotes the displacement vector, $\rho_{s}$ represents the structure density, σ is the stress tensor, and F refers to the external force term. To maintain frequency-domain consistency with Equation (4), this study adopts the harmonic assumption to convert the time term into a frequency term, which is expressed as:

(6) $$-\omega^{2}\rho_{s}u-\nabla \cdot \sigma =F$$

Therefore, both Equations (4) and (6) are frequency-domain equations, ensuring the unity of the acoustic field and the structural field within the solution domain. At the interface between the acoustic field and the solid, the coupling boundary conditions of velocity continuity and stress balance are satisfied:

(7) $$n\cdot \nabla p=-\rho_{0}\omega^{2}(n\cdot u)$$

(8) σ·n=−pn

*n* refers to the interface normal vector, and $\rho_{0}$ is the fluid density. The bidirectional coupling between sound pressure and structural vibration is realized through this condition. After finite element discretization, the coupling equation can be represented as:

(9) $$\left[\begin{array}{cc} K_{s}-\omega^{2}M_{s} & C_{as}\\ C_{as}^{T} & K_{a}-\omega^{2}M_{a} \end{array}\right]\left[\begin{array}{c} u\\ p \end{array}\right]=\left[\begin{array}{c} f_{s}\\ f_{a} \end{array}\right]$$

$K_{s}$ and $K_{a}$ are the structural and acoustic stiffness matrices, respectively; $M_{s}$ and $M_{a}$ denote the mass matrices; $C_{as}$ is the coupling matrix. This study uses a frequency-domain direct solver to iteratively solve this system, ensuring the interaction between sound pressure and structural response.

During the simulation modeling process, this study adopts a 2D planar acoustic field and solid structure coupling model to fully describe the propagation of sound waves in the fluid domain and their effect on the structure; meanwhile, it considers the feedback of structural vibration on the acoustic field. For the periodic type, the structure adopts a 2D planar periodic arrangement in the x-y plane. The unit realizes the approximation of infinite periodic expansion through periodic boundary conditions (Floquet periodic boundaries). The boundary conditions of the model are set as follows:

The incident end is set as a plane wave sound pressure source to simulate normally incident plane sound waves and ensure that sound waves propagate along the normal direction of the structure surface. The exit end is set as a non-reflective boundary to effectively avoid the interference of sound waves reflected from the exit on the simulation results. The side walls adopt hard boundary conditions to ensure the constraint of the structural transverse vibration. The air cavity inside the structure enables the “Thermoviscous Acoustics” module to consider the energy loss of sound waves in the cavity due to viscosity and heat conduction, thereby improving simulation accuracy. Damping mainly comes from two aspects. One is the internal friction damping of the material, which realizes low-frequency energy dissipation through a high-loss elastic layer; the other is the viscous and heat conduction damping in the cavity, which is simulated through the thermoviscous acoustics module.

**Figure 4:**
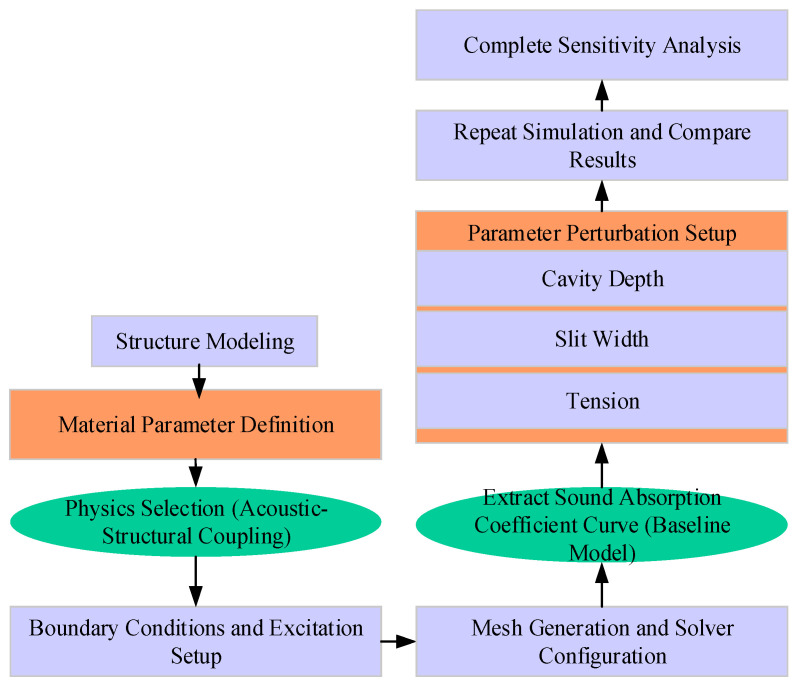
Simulation analysis flow of the underwater acoustic absorption unit structure.

The solid diaphragm material adopts a linear elastic model, and pre-stretching stress is applied in the form of boundary loads to simulate the actual vibration characteristics of the diaphragm. Finite element discretization uses second-order Lagrange elements. The mesh division ensures that the sound wave wavelength corresponding to the maximum calculation frequency is divided by at least six elements to ensure simulation accuracy. The simulation utilizes a frequency-domain direct solver with a frequency step of 10 Hz and a frequency scanning range of 200–5000 Hz, ensuring coverage of the designed target sound absorption frequency band. It is assumed that sound waves are normally incident, meaning the sound wave propagation direction is parallel to the normal of the structure’s surface. This assumption helps analyze the acoustic absorption performance of the material under standard incident conditions and facilitates the comparison and optimization of subsequent results. The simulation process is presented in Figure 4.

To investigate the regulation mechanism of multi-cavity structural parameters on acoustic absorption performance, the following key geometric and physical parameters are selected as objects for sensitivity analysis. These parameters include the depths of the main cavity and sub-cavities (H_1_, H_2_); the slit width (w); the diaphragm thickness (t) and pre-tension magnitude (T); the length of the coupling channel between multiple cavities and the opening position. In each group of parameter analysis, the remaining parameters are kept unchanged, and the value range of the target parameter is adjusted one by one. The absorption coefficient variation curves in the frequency range from 1 kHz to 5 kHz are recorded to evaluate the influence trends on the absorption peak frequency’s position, bandwidth, and peak value. Table 2 summarizes the influence trends and physical mechanisms of changes in main cavity depth, sub-cavity depth, slit width, diaphragm thickness, and pre-tension on absorption peak frequency, peak coefficient, and effective bandwidth.

Table 3 lists all key input parameters used in the finite element analysis of this study, along with their specific values and sources. Among these parameters, material properties are mainly derived from typical values in the built-in material library of COMSOL Multiphysics software, values from published literature, and standard data provided by material suppliers; all geometric parameters and physical parameters (such as pre-tension) are values set in this study based on design objectives; no external datasets or unpublished previous research results are relied on for model optimization. Table 4 lists the damping types and damping coefficients or loss factors of each layer of material in this study. They are used to describe the energy dissipation characteristics of the materials under low-frequency sound waves.

To ensure full reproducibility of the simulation, the complete set of simulation setup details has been made public. This includes a comprehensive description of the COMSOL Multiphysics model, covering the specific modules and physical interfaces used, all boundary conditions and settings, mesh information, solver configurations, and material properties. These resources are hosted in a GitHub repository (https://github.com/C3R8U/Design-and-Acoustic-Performance-Research-of-Underwater-Acoustic-Absorption-Metamaterials, accessed on 10 September 2025).

## 3. Results and Discussion

### 3.1. Analysis of Simulation Results on Acoustic Absorption Performance

**Figure 5:**
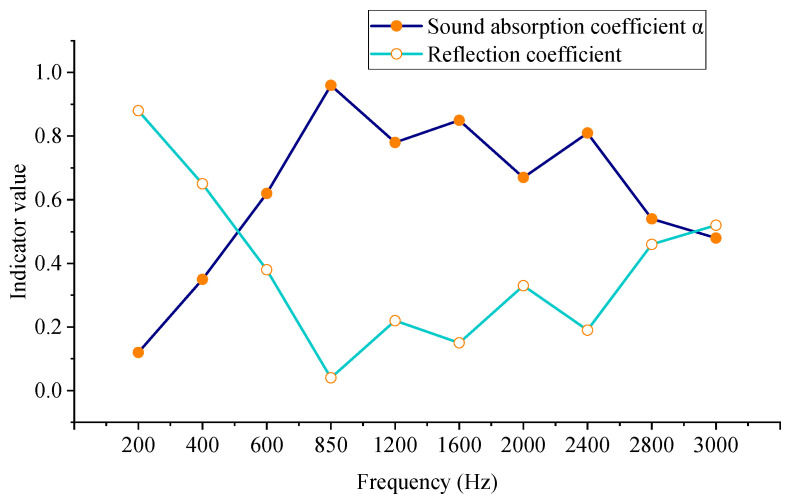
The performance at typical frequency points of the underwater acoustic absorption metamaterial structure.

Figure 5 lists the typical performance indicators of the designed underwater acoustic absorption metamaterial structure in the frequency band from 200 Hz to 3000 Hz. These indicators include the acoustic absorption coefficient, reflection coefficient, and bandwidth characteristics of key frequency bands. Simulation results show that the structure reaches the maximum absorption peak near 850 Hz, with an acoustic absorption coefficient as high as 0.96 and a reflection coefficient of only 0.04; this indicates an excellent sound wave absorption effect. At the low-frequency end of 600 Hz, the acoustic absorption coefficient exceeds 0.6, which shows that the structure can effectively activate the low-frequency acoustic absorption bandwidth, notably outperforming traditional materials.

In addition, two obvious secondary absorption peaks appear at 1600 Hz and 2400 Hz, respectively, with acoustic absorption coefficients reaching 0.85 and 0.81. This fully reflects the multi-frequency bandwidth acoustic absorption capability brought by the multi-cavity coupling design. The overall effective acoustic absorption bandwidth covers a high-efficiency absorption region in the range of approximately 600 Hz to 2800 Hz. Among them, the acoustic absorption coefficient remains above 0.5, meeting the needs of actual underwater noise control. The variation trend of the reflection coefficient is opposite to that of the acoustic absorption coefficient, with reflection dropping to its lowest at the peak frequency, further verifying the efficient dissipation of sound energy.

The acoustic absorption coefficient curve shows obvious fluctuations and sharp peaks at some frequency points, which are caused by the combined effect of the following reasons. The designed multi-cavity and multi-layer structure generates local resonance at different frequencies. Each cavity and diaphragm layer may correspond to one or more high-Q acoustic absorption peaks, making the acoustic absorption coefficient change very steeply with frequency. There is coupling between the cavities; the pre-stretching of the diaphragm and the energy dissipation of the damping layer cause slight nonlinear fluctuations in the position and amplitude of the resonance peaks, further increasing the unsmoothness of the curve. The frequency step is 10 Hz; even with a frequency-domain direct solver, it may not fully capture the continuous change of high-Q peaks, resulting in curve fluctuations at discrete frequency points. It should be emphasized that these fluctuations reflect the real physical properties of the material and structure, and do not affect the overall acoustic absorption performance analysis. The effective acoustic absorption bandwidth and peak trend remain reliable, and can be used to evaluate the acoustic absorption effect of the structure in low-frequency and wide-frequency bands. Overall, the designed structure not only achieves a strong absorption peak but also has a wide effective acoustic absorption frequency band, making it suitable for noise control in complex underwater environments.

**Figure 6:**
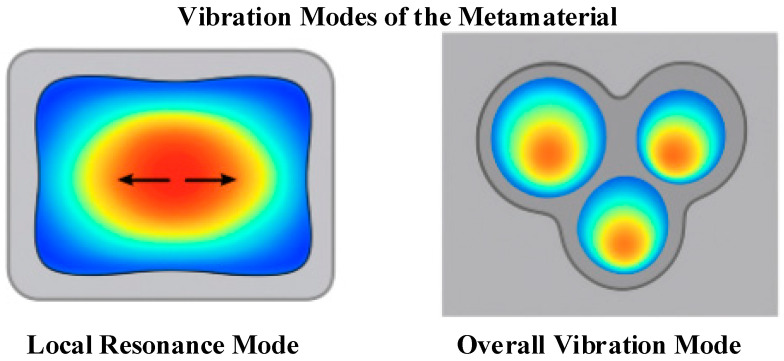
Distribution of local resonance of the flexible diaphragm and overall vibration of the multi-cavity.

To further explain the physical mechanism behind the multi-peak absorption behavior observed in Figure 5 and Figure 6 shows the vibration mode distribution of a representative multi-cavity unit at typical frequency points. Local resonance of the flexible diaphragm is clearly visible at the low-frequency peaks, indicating that vibration is concentrated in the diaphragm area. At higher frequencies, multi-cavity coupling leads to distributed vibration of the entire unit, highlighting the synergistic resonance effect. These modal patterns provide an intuitive explanation for the sudden peaks and fluctuations in the absorption coefficient curve. This confirms that the designed structure effectively utilizes local and coupled resonance for broadband underwater sound absorption. Regions with high amplitude are displayed in red, while edge regions with small amplitude are displayed in blue, showing the local resonance mode.

### 3.2. Results of the Sensitivity Analysis of Key Parameters

**Figure 7:**
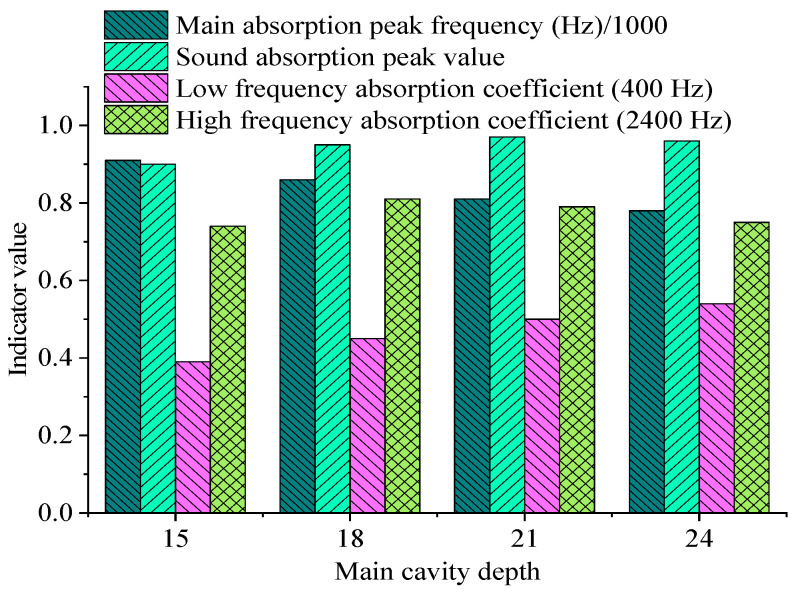
Acoustic absorption performance under different main cavity depths.

**Figure 8:**
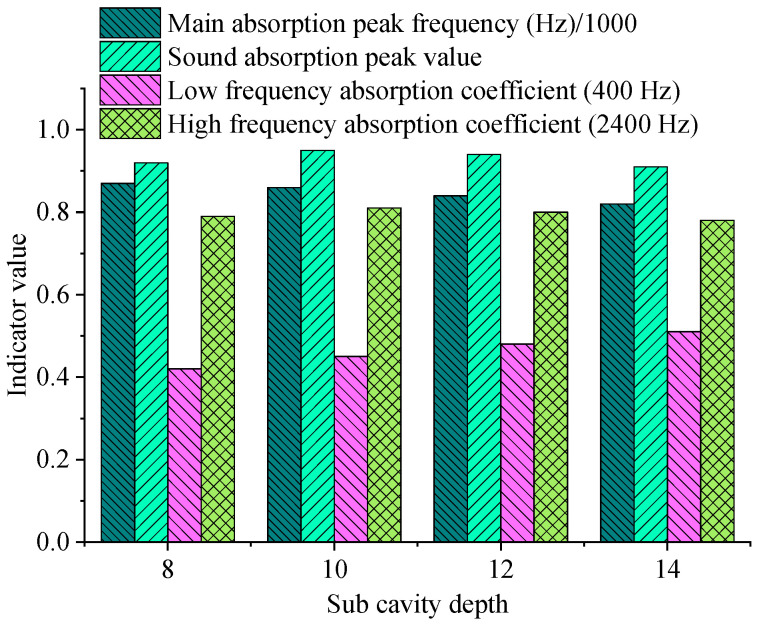
Various sub-cavity depths’ acoustic absorption performance.

To examine the influence of main cavity and sub-cavity depths on underwater acoustic absorption metamaterial performance, a series of simulations are performed by independently varying these parameters. All other design parameters remain unchanged during the analysis. The peak acoustic absorption coefficient, peak frequency, and bandwidth performance under various depth combinations are compared. The results are revealed in Figure 7 and Figure 8, and Table 5:

It can be found from Figure 7 and Figure 8, and Table 4 that the main cavity depth H_1_ substantially impacts acoustic absorption performance. As H_1_ increases from 15 mm to 24 mm, the main absorption peak frequency gradually decreases from 910 Hz to 780 Hz; this indicates that the increase in main cavity depth effectively reduces the resonance frequency, which is beneficial to broadening the acoustic absorption bandwidth in the low-frequency band. The peak fluctuates between 0.90 and 0.97, maintaining a high level overall. Additionally, the acoustic absorption coefficient at the low-frequency end is markedly improved, illustrating that the deep main cavity enhances the dissipation capacity of low-frequency sound energy.

Changes in sub-cavity depth H_2_ also have a regulatory effect on acoustic absorption performance, but the influence range is relatively small. With the increase of H_2_, the absorption peak frequency slightly decreases, and the peak value and bandwidth change little; this reflects that the tuning effect of the sub-cavity on the overall structure is more reflected in the auxiliary functions of fine-tuning resonance characteristics and improving acoustic absorption bandwidth. The acoustic absorption coefficient at the low-frequency end increases with the deepening of the sub-cavity, supporting its positive impact on low-frequency acoustic absorption. Overall, as the main resonance cavity size parameter, the main cavity depth has a notable effect on adjusting the low-frequency resonance frequency and bandwidth, and is the key to achieving low-frequency acoustic absorption broadband. As an auxiliary adjustment parameter, the sub-cavity depth further improves the structure’s acoustic absorption bandwidth and stability by refining the acoustic coupling path and optimizing the multi-cavity resonance mode.

**Figure 9:**
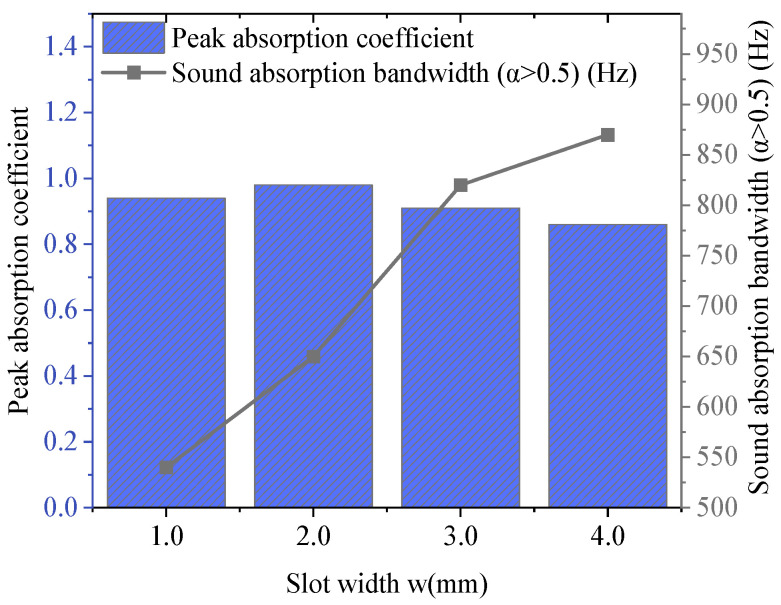
Acoustic absorption performance based on different slit width conditions.

Then, keeping other parameters unchanged and only changing the slit width w to 1.0 mm, 2.0 mm, 3.0 mm, and 4.0 mm, respectively, the corresponding acoustic absorption coefficient curves are demonstrated in Figure 9. When w = 2.0 mm, the peak acoustic absorption coefficient of the system reaches the maximum value of 0.98, showing the strongest sound energy dissipation capacity. As the slit further widens, the peak decreases slightly. This indicates that under a moderate slit width, the coupling effect between sound waves and the diaphragm is the most prominent, and the impedance matching effect is optimal. When the slit width increases from 1.0 mm to 4.0 mm, the system’s acoustic absorption bandwidth expands distinctively, from 540 Hz to 870 Hz, and the response capacity in the low-frequency band is enhanced.

**Figure 10:**
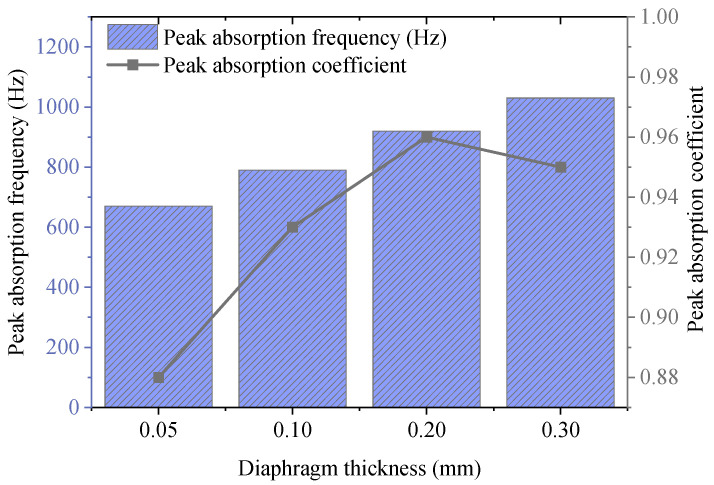
Influence of diaphragm thickness on acoustic absorption performance.

To explore the regulatory effect of diaphragm thickness *t* on acoustic absorption performance, other parameters are kept unchanged, and the results are plotted in Figure 10. It reveals that as the diaphragm thickness rises, the overall stiffness of the system improves, leading to an increase in the diaphragm’s vibration frequency and thus a shift of the absorption peak frequency to the high-frequency direction. At the same time, the increase in thickness enhances the energy dissipation capacity of the diaphragm, thereby improving the peak absorption coefficient. When the diaphragm thickness is 0.2 mm, the system achieves optimal acoustic absorption performance, with a peak coefficient of 0.96 and a bandwidth covering 760–1080 Hz. If the thickness continues to increase, the resonance frequency can be further increased. However, the acoustic absorption intensity decreases slightly, indicating that excessive thickness limits the diaphragm’s effective coupling vibration. Therefore, in actual design, the diaphragm thickness needs to be reasonably controlled to balance the acoustic absorption frequency and intensity, with a recommended thickness range of 0.1–0.2 mm.

**Figure 11:**
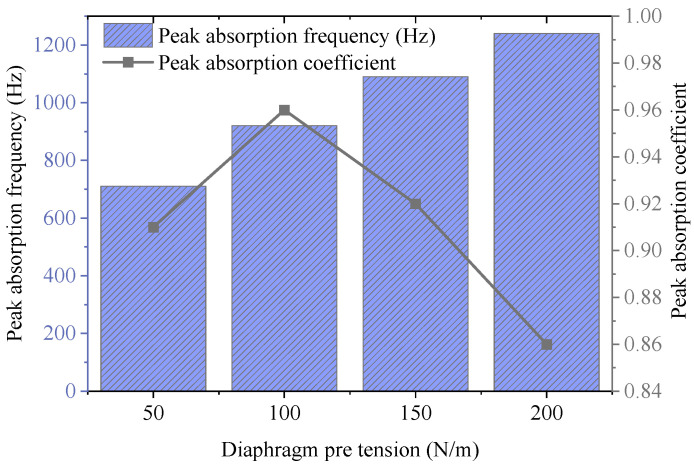
Influence of diaphragm pre-tension on acoustic absorption performance.

Then, under the condition of diaphragm thickness *t* = 0.2 mm, different pre-tension values T = 50, 100, 150, 200 N/m are set to analyze their influence on acoustic absorption performance. The results are depicted in Figure 11. The results show that the absorption peak frequency shifts markedly toward higher frequencies with increasing pre-tension. This observation aligns with the expected behavior where enhanced diaphragm stiffness elevates the vibration frequency. The acoustic absorption coefficient shows a trend of first rising and then falling. It reaches the maximum value of 0.96 when T = 100 N/m, and then decreases slightly, indicating that excessive pre-tension weakens the low-frequency response capability of the diaphragm. In addition, the bandwidth generally shifts upward with the increase of pre-tension, but its width changes little; this reveals that pre-tension mainly regulates the center frequency and has limited influence on the absorption width. Consequently, the selection of diaphragm pre-tension should balance the improvement of frequency response and the maintenance of acoustic absorption intensity, with a recommended range of 80–120 N/m.

## 4. Conclusions

This study systematically investigates the design and acoustic performance of a new type of underwater acoustic absorption metamaterial structure through finite element simulation methods. Based on the acoustic–solid coupling model established using COMSOL Multiphysics software, the study delves into the acoustic absorption characteristics and parameter regulation rules of the multi-layer periodic unit structure within the 200–3000 Hz frequency band. Simulation results show that the structure exhibits excellent low-frequency acoustic performance within a specific frequency band. Moreover, by adjusting key structural parameters such as diaphragm thickness, pre-tension, and cavity size, it can effectively control the acoustic absorption bandwidth and peak position, demonstrating good adjustability and engineering application potential. It should be noted that the established finite element model is based on a series of necessary simplifications and assumptions, which to a certain extent constitute the model’s limitations. First, the model assumes that all materials are perfectly uniform and isotropic linear elastic bodies, without considering defects, nonlinearity, or frequency-dependent characteristics that may exist in actual materials. Second, boundary conditions are idealized. For example, the side walls are set as perfectly rigid boundaries, and the outlet as a perfectly matched layer, which may differ from the complex boundary constraints in actual engineering structures. Finally, the current simulation is conducted in a static and uniform underwater environment. Potential impacts of real marine environmental factors (such as water flow impact, temperature gradient, hydrostatic pressure changes, and biological adhesion) on acoustic performance have not been comprehensively considered. Although these simplifications are conducive to exploring core mechanisms and parametric analysis, when promoting the research results to practical applications, the impacts of these factors must be investigated more in-depth.

Although this study verifies the acoustic performance of the proposed metamaterial structure through finite element simulation, its performance in the actual underwater environment still needs to be further tested through experiments. For this purpose, corresponding experimental research is planned in subsequent work. Specifically, it includes manufacturing metamaterial samples with optimized parameters; it measures their normal incidence acoustic absorption coefficients using the pulse tube method or transfer function method in an underwater acoustic duct or anechoic tank. Meanwhile, this study systematically investigates the impacts of environmental factors (such as hydrostatic pressure and temperature) on performance. Comparing experimental data with simulation results can verify the accuracy of the numerical model and provide a more reliable basis for the practical application of the structure.

## Figures and Tables

**Table 1:** Key physical and acoustic parameters of each layer of materials.

Material Layer	Material Type	Density (kg/m^3^)	Sound Velocity (m/s)	Acoustic Impedance (Pa·s/m)	Elastic Modulus (MPa)	Main Function
The panel layer	Polyurethane film	1200	1800	2.16 × 10^6^	50	Local resonance excitation, impedance matching
The damping layer	Silicone composite elastomer	1100	1400	1.54 × 10^6^	10	Vibration damping, energy dissipation
The supporting substrate layer	Fiberglass reinforced plastic	1900	2500	4.75 × 10^6^	3500	Mechanical support, multi-cavity coupling path formation
The backing rigid plate layer	Aluminum alloy	2700	5000	1.35 × 10^7^	70,000	Sound wave reflection, structural support

**Table 2:** Influence of key parameters on acoustic absorption performance.

Parameter	Trend of Parameter Changes	Changes in Absorption Peak Frequency	Changes in Peak Coefficient	Changes in Effective Bandwidth	Main Physical Mechanism
Main cavity depth	Increase	Moves toward lower frequency	Basically maintained at a high level	Widening	It increases the effective volume of the cavity, lowers the system’s resonance frequency, and enhances low-frequency sound energy dissipation.
Sub-cavity depth	Increase	Slightly moves toward a lower frequency	Little change	Slightly widening	Its auxiliary adjustment of the sound coupling path optimizes the multi-cavity resonance mode, playing a fine-tuning role in the main resonance frequency.
Slit width	Increase	Change is not obvious	First increase, then decrease	Significantly widening	Its optimization of impedance matching enhances the coupling efficiency between sound waves and the membrane; being too wide weakens local resonance effects.
Diaphragm thickness	Increase	Moves toward higher frequency	First increase, then decrease	Change is minimal	It increases diaphragm stiffness, raising the resonance frequency; at the same time, it enhances damping dissipation; being too thick suppresses effective vibration.
Diaphragm pre-tension	Increase	Moves toward higher frequency	First increase, then decrease	Slightly high-frequency movement	This is equivalent to increasing diaphragm stiffness, raising the resonance frequency; excessive tension may limit the diaphragm’s amplitude, weakening the low-frequency response.

**Table 3:** Key input parameters of the simulation model and their sources.

Parameter Category	Parameter Names and Symbols	Value	Source and Description
Geometric parameters	Element length *L*	100 mm	The design value of this study
	Total element height *H*	40 mm	
	Thickness of the panel layer t_1_	5 mm	
	Thickness of the damping layer t_2_	10 mm	
	Thickness of the substrate layer t_3_	25 mm	
	Main cavity depth *H*_1_	18 mm	
	Sub-cavity depth *H*_2_	10 mm	
	Slit width *w*	2.0 mm	
	Diaphragm thickness *t*	0.2 mm	
Material parameters	Density of the panel layer	1200 kg/m^3^	Typical values of polyurethane film [30]
	Sound speed of the panel layer	1800 m/s	
	Elastic modulus of the panel layer	50 MPa	
	Density of the damping layer	1100 kg/m^3^	Typical values of silicon-based composite materials [31]
	Sound speed of the damping layer	1400 m/s	
	Elastic modulus of the damping layer	10 MPa	
	Density of the substrate layer	1900 kg/m^3^	Typical values of fiberglass reinforced plastic
	Sound speed of the substrate layer	2500 m/s	
	Elastic modulus of the substrate layer	3.5 GPa	
	Density of the backing plate	2700 kg/m^3^	Typical values of aluminum alloys
	Sound speed of the backing plate	5000 m/s	
	Elastic modulus of the backing plate	70 GPa	
Physical parameters	Pre-tension T	100 N/m	The design value of this study
	Density of the water medium	1000 kg/m^3^	Built-in values of COMSOL
	Sound speed in a water medium	1500 m/s	

**Table 4:** Material damping parameter.

Material Layer	Material Type	Damping Type	Damping Coefficient/Loss Factor	Remarks
Panel layer	Polyurethane film	Material damping	0.02	Literature/Typical COMSOL values
Damping layer	Silicon-based composite materials	Material damping	0.10	High-loss elastomer, used for energy dissipation
Supporting substrate layer	Fiberglass reinforced plastic	Material damping	0.005	Low internal damping
Backing rigid plate	Aluminum alloys	Structural damping	0.001	Mainly provides rigid support

**Table 5:** Acoustic absorption bandwidth across diverse cavity depths (α > 0.5) (Hz).

Main Cavity Depth (mm)	Acoustic Absorption Bandwidth (α > 0.5) (Hz)	Sub-Cavity Depth (mm)	Acoustic Absorption Bandwidth (α > 0.5) (Hz)
15	700–1300	8	690–1350
18 (Benchmark value)	680–1400	10 (Benchmark value)	680–1400
21	650–1450	12	670–1420
24	620–1500	14	660–1430

## Data Availability

The original contributions presented in this study are included in the article. Further inquiries can be directed to the corresponding author.

## References

[1] Moretti P.F., Affatati A. (2023). Understanding the impact of underwater noise to preserve marine ecosystems and manage anthropogenic activities. Sustainability.

[2] Mihailov M.E. (2025). Characterization and Automated Classification of Underwater Acoustic Environments in the Western Black Sea Using Machine Learning Techniques. J. Mar. Sci. Eng..

[3] Nie W., Zhang X., Xu J., Guo L., Yan Y. (2023). Adaptive direction-of-arrival estimation using deep neural network in marine acoustic environment. IEEE Sens. J..

[4] Zhao M., Wang W., Ren Q., Ni H., Xiao X., Ma L. (2023). Modified you-only-look-once model for joint source detection and azimuth estimation in a multi-interfering underwater acoustic environment. J. Acoust. Soc. Am..

[5] Chen X., Meng L., Liu Z., Yang F., Jiang X., Yang J. (2023). Multifunctional integrated underwater sound absorption materials: A review. Appl. Sci..

[6] Sun W., Ren S., Wang Q., Che F., Lei Y., Wang H., Zhang X., Hou H., Zeng X. (2024). Underwater sound absorption characteristics of water-saturated porous materials. Eur. J. Mech.-A/Solids.

[7] Sharifi M.J., Ghalehkhondabi V., Fazlali A. (2022). Investigation of the underwater sound absorption and damping properties of polyurethane elastomer. J. Therm. Anal. Calorim..

[8] Baena J.C., Wang C., Fu Y., Kabir I.I., Yuen A.C.Y., Peng Z., Yeoh G.H. (2023). A new fabrication method of designed metamaterial based on a 3D-printed structure for underwater sound absorption applications. Appl. Acoust..

[9] Fu Y., Wang H., Cao P. (2023). Numerical design and optimization of metamaterials for underwater sound absorption at various hydrostatic pressures. J. Low Freq. Noise Vib. Act. Control.

[10] Feng Z., Xu X., Wen S., Wu Z., Li F. (2025). Enhanced sound absorption properties of a semi-open underwater periodic acoustic metamaterial. Compos. Struct..

[11] Ren S., Sun W., Zhao Z., Liu Y., Wang Q., Che F., Wang H., Lei Y., Zeng X. (2025). Underwater low-frequency sound absorption of water-saturated porous meta-material with metallic chamber. Appl. Acoust..

[12] Zhang Y., Tong M., Rui X., Wang G., Yang F., Zhou Q., Cheng L., He B. (2024). Ultrathin and pressure-resistant meta-coating with embedded tree-shaped acoustic black hole for broadband low-frequency underwater sound absorption. Appl. Acoust..

[13] Wang C., Cai L., Gao M., Jin L., Sun L., Tang X., Shi G., Zheng X., Guo C. (2024). Manufacturing of membrane acoustical metamaterials for low frequency noise reduction and control: A review. Mech. Adv. Mater. Struct..

[14] Dong L., Fan Y., Cui J., Li Y., Liu S., Zhao D. (2024). Underwater sound absorption characteristics of compliant acoustic coatings with adjustable stiffness. J. Phys. D Appl. Phys..

[15] Zarastvand M.R., Ghassabi M., Talebitooti R. (2022). Prediction of acoustic wave transmission features of the multilayered plate constructions: A review. J. Sandw. Struct. Mater..

[16] Wu L., Zhang X., Ban J., Jiang Q., Li T.-T., Lin J.-H., Tang Y. (2021). Design and optimization of multi-scale porous sandwich composites with excellent sound absorption and cushioning properties. J. Sandw. Struct. Mater..

[17] Yang X., Li Q., Shen X., Zhou B., Wang N., Wang E., Zhang X., Shen C., Wang H., Jiang S. (2025). Interlayer Parallel Connection of Multiple Helmholtz Resonators for Optional Broadband Low Frequency Sound Absorption. Materials.

[18] Jang J.Y., Song K. (2023). Synergistic acoustic metamaterial for soundproofing: Combining membrane and locally resonant structure. Int. J. Mech. Sci..

[19] Xu J., Ning D., Chen L., Liu H.-W. (2024). Numerical investigation on local resonance within an array of C-shaped cylinders in water waves. J. Hydrodyn..

[20] Xie S., Li Z., Yan H., Yang S. (2022). Ultra-broadband sound absorption performance of a multi-cavity composite structure filled with polyurethane. Appl. Acoust..

[21] Li Z., Fan G., Zhao J., Zeng X., Yan C. (2025). Study on the fluid cross-mixing characteristics in tube bundle channels with transverse uneven heat flux distribution. Energy.

[22] Siriboon J., Magaraphan R. (2025). Devulcanization and functionalization of ground tire rubber for the novel metal sheet roof with strong sound absorber and thermal insulation. Clean. Eng. Technol..

[23] Zeng X., Li G., Zhu J., Sain M., Jian R. (2023). NBR/CR-Based High-Damping Rubber Composites Containing Multiscale Structures for Tailoring Sound Insulation. Macromol. Mater. Eng..

[24] Nakayama M. (2024). Acoustic metamaterials based on polymer sheets: From material design to applications as sound insulators and vibration dampers. Polym. J..

[25] Liao G., Luan C., Wang Z., Liu J., Yao X., Fu J. (2021). Acoustic metamaterials: A review of theories, structures, fabrication approaches, and applications. Adv. Mater. Technol..

[26] Dong E., Cao P., Zhang J., Zhang S., Fang N.X., Zhang Y. (2023). Underwater acoustic metamaterials. Natl. Sci. Rev..

[27] Failla G., Marzani A., Palermo A., Russillo A.F., Colquitt D. (2024). Current developments in elastic and acoustic metamaterials science. Philos. Trans. A.

[28] Xu J., Cai H., Wu Z., Li X., Tian C., Ao Z., Niu V.C., Xiao X., Jiang L., Khodoun M. (2023). Acoustic metamaterials-driven transdermal drug delivery for rapid and on-demand management of acute disease. Nat. Commun..

[29] Comandini G., Ouisse M., Ting V.P., Scarpa F. (2025). Architected acoustic metamaterials: An integrated design perspective. Appl. Phys. Rev..

[30] Wang T., Wang G.B., Zhang R.J., Ke M.-Z. (2022). Low-frequency underwater sound absorption metamaterial. Phys. Scr..

[31] Martínez J.A.I., Farhat M., Wu Y., Khelif A. (2025). Ultrasound underwater coherent perfect absorbers. Phys. Rev. Appl..

